# Supplementary material for: A Hybrid Type II Hub-and-Spoke Model Evaluation Framework in the Commonwealth Partnerships for Antimicrobial Stewardship Programme—A Study Protocol
Source: Antibiotics (Basel). 2025 Dec 3;14(12):1218. doi: 10.3390/antibiotics14121218 (PMC12729711; doi:10.3390/antibiotics14121218)
Supplement: Supplementary file 1 [file antibiotics-14-01218-s001.zip › antibiotics-3924489-supplementary.pdf]

Title:

A Hybrid type II hub-and-spoke model evaluation framework in CwPAMS 2.0

Appendix 1:

CwPAMS Antimicrobial Stewardship Assessment Tool

The Commonwealth Partnerships for Antimicrobial Stewardship (CwPAMS) AMS assessment tool is based on the WHO periodic healthcare facility assessment tool and practical toolkit for *Antimicrobial Stewardship Programmes in HealthCare Facilities in Low- And- Middle-Income Countries*. It is provided to prospective CwPAMS partnerships to ensure uniform and evidence-based gathering of baseline data on the state of antimicrobial consumption surveillance and antimicrobial stewardship (AMS), as well as barriers and facilitators for implementing AMS interventions within facilities. The assessment, in addition to any other scoping work completed by the partnership teams, should support the application proposal and subsequent AMS action plan developed during project implementation.

Responses may be gathered from desk reviews, individual or group interviews, observations in healthcare facilities, focus group discussions and data already available through healthcare facility records and documents. In most cases, a desk-based approach is sufficient.

The Tool comprises the following main sections

- Identification section for provision of facility and partnership details
- The healthcare facility assessment section, exploring status of the core elements of antimicrobial stewardship: Leadership Commitment; Accountability and Responsibility; AMS Actions; Education and Training; Monitoring and Surveillance; Reporting Feedback within Healthcare facility
- Analysis of opportunities and threats following the above assessment
- Healthcare workforce capacity in terms of available staff and their responsibilities (Pharmacists, Nurses, microbiologists, medical doctors, among others)

At the end of the programme, a post-AMS assessment form is completed to assess the impact of the interventions

Appendix 2:

CwPAMS 2 Narrative Report

The intention of this report is to capture all the work the CwPAMS health partnerships (HPs) are doing outside of the quantitative MEL plan and identify areas where the programme management can support the HPs further. As such the focus is on qualitative responses, as the MEL portal captures quantitative responses. HPs are expected to complete this form on a bi-annual basis within the programme timeline (March 2023–March 2025).

The form collects information on the following

- Identification details of the health partnership team who have participated in filling in the form
- Work plan progress and challenges/changes encountered
- Key Projects highlights
- Awareness activities and training conducted in the following areas: AMS, IPC, antimicrobial use surveillance, quality improvement, one health, integration and use of clinical microbiology data, and substandard and falsified medicines
- Data sharing and learning opportunities within healthcare facilities and at national level
- A special section for Hub-and-spoke partnerships to detail activities implemented in spokes and any challenges, barriers and facilitators observed working as a network
- Utilised AMR tools and resources
- GESI

- Communication and Policy influence
- Finance management as part of programme management

Appendix 3:

RE-AIM Post implementation evaluation Quantitative survey constructs

Dimensions	Items
**Reach**<br>Reach refers to the absolute number, proportion, and representativeness of individuals who were willing to participate in a given initiative, intervention, or program	
Exclusion Criteria (percentage and characteristics)	1) How many potential participants were approached?
	2) How many participants were deemed ineligible to participate?
	3) Of the participants meeting selection criteria, how many participants actually participated?
Percentage on the Valid Denominator	4) Was the percentage of all eligible invited participants who accepted participation reported? If not, can the percentage be calculated?
Characteristics of Participants Compared with Nonparticipants	5) Were the characteristics of those subjects choosing to participate and those unwilling to participate described? If yes, what was the representativeness of those who participated versus those who did not? Comparisons can be made with either nonparticipants or available resource data.
**Effectiveness**<br>Effectiveness refers to the impact of an intervention on important outcomes, including potential negative effects, quality of life, and economic outcomes.	
What are the most important outcomes?	6. a) Was the targeted individual/organizational change? Did it reach, what it meant out to reach?<br>6.b) Any potential behaviour-level change which was targeted, achieved?<br>6.c) Any guidelines/policies development/revisions which was targeted, was it achieved?
Measure of the Effect Size	7) If applicable- were effect sizes for significant outcomes provided or can they be calculated?
Measure of Robustness Across Subgroups	8) Were any within-groups analyses conducted that allowed researchers to draw conclusions about how different subpopulations (spokes) demonstrate effectiveness?
**Adoption**<br>Adoption refers to the absolute number, proportion, and representativeness of settings and intervention agents (people who deliver the program) who are willing to initiate a program.	
Fidelity to the implementation plan (hub)	9) How many people in the hubs participated in the program as intended?
	10) How many times did the implementation plan of the HSM spoke lead to change?
Fidelity to implementation plan<br>(how many people adapted the target	11) What percentage of the total number of people actually used the interventions/actions delivered through HSM?

Dimensions	Items
outcome/output) at each hub	
Characteristics of Participants who adapted the intervention/innovation Compared with Nonparticipants	12) Were the characteristics of those settings choosing to participate and those unwilling to participate described? If yes, what was the representativeness of those that participated versus those that did not? Comparisons can be made with either nonparticipating settings or available resource data.
**Implementation**<br>At the setting level, implementation refers to the intervention agents' fidelity to the various elements of an intervention's protocol, including consistency of delivery as intended and the time and cost of the intervention. At the individual level, implementation refers to clients' use of the intervention strategies.	
Percent of Perfect Delivery (Implementation Fidelity)	15) Were there measures (preferably statistical but also observational) indicating that Agent A and Agent B or training A and Training B delivered the same amount/type of intervention/both successful?
	16) Were the methods of intervention delivery described?
	17) Were there any measures of the actual number of intervention pieces that were delivered (e.g., an average or percentage)?
Adaptation (to intervention)	18) Was the intervention/implementation plan changed or modified in any way during the course of the study?
Cost of Intervention	19) Was any information provided on the total cost required to implement the intervention?
	20) Was any information provided on the type of staff needed to implement the intervention?
	21) Was any cost-effectiveness or cost-benefit analysis done to determine the economic soundness of the intervention?

Appendix 4:

Qualitative interviews/focus group discussions:

Implementation process evaluation of the HSM model:

Reach:

How did you hear about this HSM program?

Probe: through [specific organization name], flyer, friends, relatives

What made you interested in joining? (e.g., what information attracted you to the program)

Prompts: group class, the training (content) type, offered through [specific organization name], mode of training, externa;/internal influencers

What did you think the HSM program could help you accomplish? -

Prompts: In case the interventions are individually focused

a. **Probe:** Improve overall personal health, manage chronic disease, improve functional fitness, a doctor recommended [the program], reach fitness goals, weight goals, etc.

What did you think the HSM program could help your organization accomplish? -

Prompts: In case the interventions are scaled to introduce a change on organizational level focused

Note: Ask both in case there are outputs (individual, organizational) and outcomes (individual, organizational)

What do you think were the best ways to tell people about the program?

a. **Probe:** Community flyers, doctor referral, phone calls, email, etc.

Where else could it have been advertised?

b. Prompts: What should be included in the (type)?

e.g., how should a physician notify? What should be on flyers

Retention:

What parts of the HSM program helped you stay involved from the beginning to the end of the most?

Probe: group class, the training (content) type, offered through [specific organization name], mode of training, externa;/internal influencers

Can you provide a few details as to why you feel this way?

What changes to the HSM program could have helped you stay more involved?

Probe: group structure, duration, benefits of attending, number of meeting sessions, location.

Can you provide a few details as to why you feel this way?

Effectiveness:

What are some of the ways HSM program has had a positive impact in your personal and/or professional life?

Please share any ways it had a negative impact (if applicable)

Adoption:

HSM program was led by [instructor type/hub name]. Who do you think should lead the program sessions in the future?

Probe: your local mentors, another Centre of excellence having more expertise, experts from national international centers

Implementation:

The HSM program was delivered [share instances, e.g., in person/virtual 2 hours once per week for 4 weeks]. How do you think it should be delivered in the future?

Frequency? For how long? In person, via web conference?

How did you feel about the cost of participating in the HSM program?

Probe: Was it useful to spend your money/time on it?

Maintenance:

Now that HSM program is over, what, if anything, are you doing to insert desired/ outcome, e.g., a behavior target?

Exploration:

Is there anything else you would like to share with the research team at this time?

Appendix 5:

Qualitative interviews/focus group discussions:

A. Awareness and initial perception

- What prior knowledge or experience do you have in using the Hub-and-spoke Model (HSM) in general, and how does this influence your impressions of its use within the CwPAMS for AMS interventions? If not, what were your initial thoughts of using it in CwPAMS 2.0?
- How did you familiarize yourself with the proposed HSM for CwPAMS? Probe more for internal training, meetings to get people on the same page (we are looking for similar/different mental models and approaches of GM/CPA leads: project management focused/ output focused, etc.)

B. Implementation and infrastructure

- Has the implementation of the HSM affected organizational infrastructure within your partnerships (they are GM, ICCs of)? If yes, how so, or what have been the changes?
- How has the hub-and-spoke model been socialized and mobilized within your partnerships?
- To what extent is the HSM being implemented as intended in the original plan?
- What are the key barriers and facilitators in implementing the model? (Consider using the socio-ecological model: individual, communal, societal, environmental, organizational, policy.)
- If there are deviations from the overall plan (CPA/THET) for HSM, why have they occurred, and what can be done to address them?

C. Stakeholder engagement, acceptability and resource allocation

- How has the HSM influenced stakeholder engagement across different levels within the HP (e.g., providers, both internal, e.g., healthcare professionals, pharmacists etc., managers, policy makers, line managers, and external, e.g., with the UK team, with FF labs, with community partners)?
- What is the general perception of the HSM among stakeholders? Is it considered acceptable and relevant/functional?
- How has the model affected communication and collaboration among stakeholders?
- Do the HPs you are looking after have the resources, staff, and equipment needed for implementation of HSM/intervention through HSM?
- Do hubs and spokes have the required time, skills, training, and ability to implement the model effectively?
-

D. Training and capacity building

What training initiatives have been implemented as part of the HSM, and how effective have they been?

Do staff have the necessary skills, time, and resources to implement the HSM effectively?

How has the model influenced capacity building within your organization?

E. Data collection, analysis, and interpretation

- How has the HSM impacted data collection, analysis, and interpretation processes?
- What changes have been made to data management practices as a result of the model?
- How are data being used to inform decision-making and measure the effectiveness of the HSM?

F. Adoption and utilization

- To what extent has the HSM been adopted by implementers within your partnerships? (Why?)
- What factors have influenced the adoption and usage of the model? Also need to consider if there are any barriers to lack of coordination/dissemination
- How has the HSM been adapted to fit different settings and technical workstreams (e.g., AMS/AMR, IPC, Microbiology, One Health, Community behavior change)?

G. Feasibility and reach

- How feasible is the implementation of the HSM in your specific HP organizational context?
- How do you track the reach and effectiveness of the HSM?

H. Effectiveness and impact

- What factors have contributed to or hindered the success of the model so far? (Why?)
- How do you measure changes and outcomes, and what threats exist to the achieving of desired results?

I. Implementation cost and sustainability

- What is the cost-effectiveness of the HSM compared to previous CwPAMS iterations?
- How has the model impacted financial resources and sustainability planning?
- Can the HSM be sustained over time within your HPs, and what infrastructure is in place to support this?

J. Feedback and continuous improvement

- Have you had opportunities to provide feedback on the HSM?
- Are there any changes you would suggest to improve the model's design, usability, or integration?
- What additional support or resources would enhance the implementation and impact of the HSM?
- What sort of feedback are you receiving from your partnerships on the implementation of HSM?

K. Technical work streams and behavior change (on hold)

- How has the HSM impacted technical work streams, such as AMS/AMR, IPC, Microbiology, and One Health?
- How does the model support or hinder behavior change initiatives? (on hold)

L. Additional insights

- Is there anything else you would like to add about the implementation of the HSM on your partnerships or the CwPAMS 2.0?

Implementation evaluation question: HP specific questions (to be asked during HSM strengthening visits)

- To what extent are the intervention/key actions being implemented as intended in the original plan?

- How is the program being implemented? What are the key barriers and facilitators regarding implementation of interventions?
- How are you measuring if the program is being implemented correctly?
- Are participants being reached as intended?
- Are interventions delivered as planned?
- Are interventions carried out as planned?
- Does the organization have the resources, staff and equipment to implement the intervention?
- Do staff have the time, skills, training and ability to implement the intervention?
- How satisfied are program managers and the intended target audience?
- What has been done differently to the original plan, and why?
- What are the key strategies to help implement the interventions?

Appendix 6:

Quantitative data collection instrument 1: Action Plan Template

Commonwealth Partnerships for Antimicrobial Stewardship (CwPAMS)

Antimicrobial Stewardship Action Plan Template

CwPAMS is funded by the Department of Health and Social Care using UK aid funding and is managed by the Fleming Fund. The Department of Health and Social Care (DHSC)'s Fleming Fund is a UK aid programme supporting up to 25 countries across Africa and Asia to tackle antimicrobial resistance (AMR), a leading public health threat across the world. The Fleming Fund invests in strengthening surveillance systems through a portfolio of country grants, regional grants and fellowships managed by Mott MacDonald, and global projects managed by DHSC partners. The CwPAMS programme is managed by the Commonwealth Pharmacists' Association and the Tropical Health Education Trust (THET).

CwPAMS Antimicrobial Stewardship Action Plan template

This tool has been designed to be used by the CwPAMS partnership teams to develop an AMS Action Plan, which is based on the assessment of AMS activities and capacity within the partnership institutions. The AMS Action Plan

allows partners to establish local priorities for the development of AMS interventions, ensuring that realistic time frames are agreed.

How to complete the action plan:

1. The Healthcare facility detailed situation analyses and the assessment tool which is completed at the start of the project should be added to section 4 Annex. Re-consider this analysis and discuss as a partnership the main priorities.
2. Partnerships should consider opportunities and threats to the locally agreed actions as part of this process.
3. The overall facility-specific action plan should be devised in discussion with relevant stakeholders and considering possible evidenced actions.
4. We recommend that you use the Change Exchange/THET/ CPA Behavioral Science Toolkit for AMS when formulating this Action Plan, with a focus on sections 3 and 4 *"Linking outcomes, behaviors and influences in a theory of change" and "Designing training to change behaviors".*
5. The AMS action plan should be agreed locally within the healthcare institution and presented at a hospital-wide committee e.g., Medicines and Therapeutics Committee (MTC), who should take responsibility for overseeing the delivery of the agreed actions.
6. Consideration should be given to the sustainability of AMS actions developed by the facility.

The Healthcare facility detailed situation analyses and assessment tool are based around the CwPAMS AMS checklist, which all CwPAMS partnerships should complete at the start of the project. This template can be used to prepare summary documents for partner institutions and can also be used for the CwPAMS Action Plan feedback, which is mandatory for each partnership.

If you have any queries, contact the Commonwealth Pharmacists' Association via cwpams2@commonwealthpharmacy.org

**Contents**	**Page number**
1. Facility and Partnership details	3
2. Executive summary	3
3. Facility specific AMS Action plan	4
4. Annex – completed Healthcare facility detailed situation analyses and assessment tool	6

**1. Facility and partnership details**	
**Date of completion**	
**Country**	
**Name of Partnership**	
**Name of the LMIC delivery healthcare facility and location this action plan is for**	

**Name of lead person completing assessment from the partnership team at the LMIC hospital institution:**	
**Date that the Action Plan was presented to the LMIC hospital institution and details of the committee it was discussed at, e.g., Medicines and Therapeutics Committee.**	

**2. Executive summary**
An abstract of the main aspects of the document

**3. Sustainability**
Detail how sustainability for AMS has been considered throughout this Action Plan.

**4. Facility specific Action Plan**
Use the table provided to formulate a summary of the principal actions (identified in the Healthcare facility detailed situation analyses and assessment tool), which will be taken forward by the organization.

Objective	Identified gaps	Action(s) agreed<br><br>How will this be done ?	Who is responsible for the Action?<br><br>(position and department)	Agreed timeframe to complete	Progress to date
**Leadership Commitment**					

**Accountability and responsibility**					
**AMS Actions**					
**Education and training**					
**Monitoring and surveillance**					
**Reporting feedback within the Healthcare facility**					

5. Annex - Healthcare Facility situation analyses and assessment tool.

Healthcare facility to insert the completed AMS checklist, which was completed at the start of the CwPAMS project.

Quantitative data collection instrument 2: AMS Pre-Post assessment tool

Commonwealth Partnerships for Antimicrobial Stewardship (CwPAMS)

Antimicrobial Stewardship (AMS) Assessment Tool

(Pre- and post-CwPAMS Intervention)

CwPAMS is funded by the Department of Health and Social Care's Fleming Fund. The Department of Health and Social Care's Fleming Fund is a UK aid programme supporting up to 25 countries across Africa and Asia to tackle antimicrobial resistance (AMR), a leading public health threat across the world. The CwPAMS programme is managed by the Commonwealth Pharmacists' Association and the Tropical Health and Education Trust (THET).

The AMS Assessment Tool

The CwPAMS AMS assessment tool is based on the WHO Practical Toolkit and provides a contextualized checklist of core AMS elements and key behaviors for use within healthcare settings in Sub-Saharan Africa. An AMS programme in a large tertiary hospital with different specialties will usually be larger and more complex than one in a district hospital. It is therefore important that healthcare facility management and an AMS committee and/or AMS team decide together which strategies best fit their local setting, based on a situational analysis. Periodic assessment using this tool, in addition to the project implementation, should inform subsequent AMS action plans. Completing all sections of this tool is highly recommended, in order to support you in considering all AMS aspects when identifying priority areas and gaps.

How to complete this form

In the true spirit of partnership, the questions in this form should be completed jointly by partners in the target country and in the UK. A **separate CwPAMS AMS assessment tool** should be completed for **each LMIC healthcare facility** involved in the CwPAMS programme. For example, if a health partnership is working with two LMIC hospitals, then two separate forms should be submitted. Some questions may not be applicable to lower-level health centres, in which case please answer these questions as "not applicable" (see guidance on page 3). **This tool is not suitable for community pharmacies.** To review progress, the form should be completed at the end of the CwPAMS programme, during or after the final country visit (or intervention).

Responses may be gathered from desk reviews, individual or group interviews, observations in healthcare facilities, focus group discussions, and data already available through healthcare facility records and documents. This may also be discussed during an AMS committee meeting. In most cases, a desk-based approach is sufficient. From experience, completing the assessment tool should take less than 2 hours.

A **verifier section** has been provided to serve as a **supportive guide, not a requirement**.

Please answer as honestly and completely as possible; there are no right or wrong answers. This assessment tool is designed to support partnerships in order to maximise their impact, NOT to assess them. Summary findings with acknowledgment of contributors will be included for programmatic level dissemination, reporting and publications. All information will be kept confidential and individual institutions will not be identified.

Completing each section of the form

To complete the assessment tool, the user should provide one response per question based on the following options.

- **Yes** - Fully implemented - the core element is in place and is fully implemented without requiring strengthening, but needing to be sustained.
- **No** - Not currently in place/implemented; activities planned but not started - the core element is planned, but no action has taken place.
- **Somewhat** - Partially implemented - the core element is in place, but it is only partially implemented, requiring further strengthening.
- **Unknown** - you do not know the answer (it is fine to select this option if you do not know or are not able to easily find out the answer).

- **Not applicable** - where a statement is not applicable within the setting, e.g., where there is no action plan, having financial resources would not be applicable.
- **Statement is not clear/**not understood.

Please use the comments column to provide additional information, comments, relevant examples or links to published documents.

Submit the completed form as an attachment to the partnership reporting.

If you have any queries, contact cwpams2@commonwealthpharmacy.org.

**Name and code of Health Partnership:**	
**Date of completion:**	
**Name of the LMIC healthcare facility and location this assessment is for. NB, Each site will require its own completed form:**	
**For Category A Health Partnerships only**	Hub ☐ Spoke ☐
**How would you describe the healthcare facility?**	Teaching Hospital ☐ District general hospital ☐<br>Regional referral hospital ☐ Lower-level health center ☐<br>Other (Please specify) ....
**Number of inpatient beds:**	
**Does this facility serve out-patients?**	Yes ☐ No ☐
**Name and email address of the lead person completing the assessment from the partnership team at the LMIC hospital institution:**	
**Name and email address of the lead person completing the assessment from the UK partnership team:**	

Healthcare Facility Assessment Tool

		**Answer**	**Verifiers**	**Comments/Additional information**
**Leadership Commitment**				
**1**	Is AMS identified as a priority by the healthcare facility/management/leadership?	Yes ☐	*Interviews with healthcare*	

	The facility management has formally identified AMS as a priority objective/	No ☐<br>Somewhat ☐<br>Unknown ☐<br>Not applicable ☐<br>Do not understand the question ☐	*facility leadership*	
**1. a**	Are AMS activities included in the healthcare facility annual plans with key performance indicators?	Yes ☐<br>No ☐<br>Somewhat ☐<br>Unknown ☐<br>Not applicable ☐<br>Do not understand the question ☐	*Annual plans of healthcare facility with AMS performance indicators*	
**1. b**	Has the healthcare facility management allocated human and financial resources to **support AMS activities?** *There is dedicated, sustainable and budgeted financial support for AMS activities, e.g., support for salary, training, and information technology (IT) support*	Yes ☐<br>No ☐<br>Somewhat ☐<br>Unknown ☐<br>Not applicable ☐<br>Do not understand the question ☐		
		**Answer**	**Verifiers**	**Comments/Additional information**

**2**	Is there a written healthcare facility AMS action plan that has been developed and approved?	Yes ☐<br>No ☐<br>Somewhat ☐<br>Unknown ☐<br>Not applicable ☐<br>Do not understand the question ☐	*Healthcare facility AMS action plan and progress report*	*Please state when this was approved/last reviewed*
**2. a**	Is there a mechanism to regularly monitor and measure the implementation of the AMS activities captured in the AMS action plan?	Yes ☐<br>No ☐<br>Somewhat ☐<br>Unknown ☐<br>Not applicable ☐<br>Do not understand the question ☐	*Regular progress report(s) on the implementation of the healthcare facility AMS action plan*	*Please indicate below the mechanism and frequency of implementation*

**Accountability and Responsibility**				
		**Answer**	**Verifiers**	**Comments/Additional information**
**3**	Is there a multidisciplinary AMS committee in the healthcare facility?<br>*This AMS committee can either be stand-alone or embedded in another existing committee structure (e.g., drug and therapeutics committee).*	Yes ☐<br>No ☐<br>Somewhat ☐<br>Unknown ☐	*AMS committee TOR*	*If yes, please indicate if:*<br>• AMS Committee Stand-alone ☐<br>• AMS embedded as part of an agenda item for another committee ☐<br>State which committee ………………………………………………………

		Not applicable ☐<br>Do not understand the question ☐		………………………………………………………………<br>……
**3. a**	Are there clear terms of reference for the AMS committee?<br>*The AMS committee is explicitly in charge of setting and coordinating the AMS programme /strategy according to its terms of reference. If embedded in another committee, AMS must be a standing item on the committee's agenda.*	Yes ☐<br>No ☐<br>Somewhat ☐<br>Unknown ☐<br>Not applicable ☐<br>Do not understand the question ☐	*AMS committee TOR*	
**3. b**	Does the AMS committee meet on a regular basis?	Yes ☐<br>No ☐<br>Somewhat ☐<br>Unknown ☐<br>Not applicable ☐<br>Do not understand the question ☐	*AMS committee TOR; AMS Committee meeting dates*	*If yes, please indicate the frequency below:*
		**Answer**	**Verifiers**	**Comments/Additional information**

**4.**	Is there an AMS **team?**<br><br>*An AMS team is a team of multidisciplinary healthcare professionals who implement the* ***day-to-day AMS activities*** *in the healthcare facility. The composition of the AMS team is flexible.*<br><br>*In resource-limited settings or small facilities it is often difficult to have an AMS team, and an AMS champion can be identified instead (see below).*	Yes ☐<br>No ☐<br>Somewhat ☐<br>Unknown ☐<br>Not applicable ☐<br>Do not understand the question ☐		
**4. a**	Do the members of the AMS **team** have dedicated staff time and clearly defined roles and responsibilities in their job descriptions for AMS activities?	Yes ☐<br>No ☐<br>Somewhat ☐<br>Unknown ☐<br>Not applicable ☐<br>Do not understand the question ☐	*Job descriptions includes AMS activities*	
**4. b**	Does the AMS **team** meet on a regular basis?	Yes ☐<br>No ☐<br>Somewhat ☐<br>Unknown ☐<br>Not applicable ☐		

		Do not understand the question ☐		
		**Answer**	**Verifiers**	**Comments/Additional information**
**5**	Is there a dedicated AMS leader/champion identified for the healthcare facility?<br>*A healthcare professional has been identified as a leader /champion for AMS activities at the facility and is responsible for implementing the AMS programme.*	Yes ☐<br>No ☐<br>Somewhat ☐<br>Unknown ☐<br>Not applicable ☐<br>Do not understand the question ☐	*AMS champion identified*	
**5. a**	Does the AMS leader/champion have dedicated staff time for AMS activity in their job description?	Yes ☐<br>No ☐<br>Somewhat ☐<br>Unknown ☐<br>Not applicable ☐<br>Do not understand the question ☐	*Job description includes AMS activities*	
**6.**	Who are the members of the AMS **committee/ team/champions**? Tick all that apply	• Pharmacists ☐		

		• Nurses ☐<br>• Medical doctors ☐<br>• Infectious diseases doctors ☐<br>• Surgeons ☐<br>• Clinical microbiologists ☐<br>• Laboratory Microbiologists ☐<br>• Laboratory scientists ☐<br>• ITU Consultants ☐<br>• Data analysts ☐<br>• Infection control staff ☐		
		**Answer**	**Verifiers**	**Comments/Additional information**
**7**	Does the AMS **committee /team /champion** collaborate with other healthcare teams such as Drug and Therapeutics, IPC, HIV/TB, and	Yes ☐<br>No ☐	*A document to show evidence of collaboratio*	

	Quality Improvement teams at the healthcare facility?	Somewhat ☐<br>Unknown ☐<br>Not applicable ☐<br>Do not understand the question ☐	*n or joint meetings*	
**8**	Does the AMS **team/committee /champion** produce regular (descriptive) activity reports on the implementation of the AMS programme/facility action plan?	Yes ☐<br>No ☐<br>Somewhat ☐<br>Unknown ☐<br>Not applicable ☐<br>Do not understand the question ☐	*AMS committee report*	
**8. a**	Is the AMS activity report disseminated to the facility management, other healthcare facility personnel and appropriate national authority?	Yes ☐<br>No ☐<br>Somewhat ☐<br>Unknown ☐<br>Not applicable ☐<br>Do not understand the question ☐	*Latest AMS activity report, summary report to national AMS TWG*	
**AMS Actions**				

		Answer	Verifiers	Comments/Additional information
**9**	Is there a standard treatment guideline for infection management at the healthcare facility? *Evidence-based guidelines using susceptibility patterns (where possible), to assist with antimicrobial selection for common clinical conditions (indication, agent, dose, route, interval, duration)*	Yes ☐<br>No ☐<br>Somewhat ☐<br>Unknown ☐<br>Not applicable ☐<br>Do not understand the question ☐	*Healthcare facility standard treatment guideline including IPC managemen t guidelines*	
**9. a**	Are the guidelines reviewed and updated periodically based on the availability of new evidence?	Yes ☐<br>No ☐<br>Somewhat ☐<br>Unknown ☐<br>Not applicable ☐<br>Do not understand the question ☐	*Document of guideline review processes*	
**9. b**	Have you developed/revised/ adopted any AMS or IPC guidelines/tools/protocols into your healthcare facility over the last year?	Yes ☐<br>No ☐<br>Somewhat ☐<br>Unknown ☐	*Please list guidelines/t ools/protoco ls*	

		Not applicable ☐<br>Do not understand the question ☐		
		**Answer**	**Verifiers**	**Comments/Additional information**
**9. c**	If yes to the above, please provide details of your implementation plans for using the AMS/IPC guidelines/tools/protocols effectively.	N/A		
**10**	Is there a regular review/audit of **specified antimicrobial therapy** or clinical conditions at the healthcare facility?<br>*Depending on available resources, this can be conducted by prioritizing wards or specific patient conditions.*	Yes ☐<br>No ☐<br>Somewhat ☐<br>Unknown ☐<br>Not applicable ☐<br>Do not understand the question ☐	*AMS audit report*	
**11**	Is the advice/feedback from AMS teams or local AMS champions easily accessible/available to prescribers?<br>*This can be achieved through various methods, including facility ward rounds, bedside consultations, and dedicated telephone lines.*	Yes ☐<br>No ☐<br>Somewhat ☐<br>Unknown ☐<br>Not applicable ☐	*Feedback report from AMS team*	

		Do not understand the question ☐		
12	Does the AMS team or local AMS champion conduct regular ward rounds and other AMS interventions in selected departments in the healthcare facility?	Yes ☐<br>No ☐<br>Somewhat ☐<br>Unknown ☐<br>Not applicable ☐<br>Do not understand the question ☐	*Ward round reports*	
		**Answer**	**Verifiers**	**Comments/Additional information**
13	Does the healthcare facility have a formulary/list of approved antimicrobials for use based on the national formulary?<br>*Approved* antimicrobials *may be based on national recommendations or the WHO Essential Medicines List.*	Yes ☐<br>No ☐<br>Somewhat ☐<br>Unknown ☐<br>Not applicable ☐<br>Do not understand the question ☐	*Healthcare facility formulary/ drug bulletin report*	
13.a	Does the healthcare facility formulary specify lists of restricted antimicrobials that require approval by a designated team or person (pre-authorization)?	Yes ☐<br>No ☐<br>Somewhat ☐	*Healthcare facility formulary with restrictions*	

		Unknown ☐<br>Not applicable ☐<br>Do not understand the question ☐		
**14**	Does the healthcare facility have access to laboratory and imaging services (on-site or off-site) that can be used to support AMS interventions?	Yes ☐<br>No ☐<br>Somewhat ☐<br>Unknown ☐<br>Not applicable ☐<br>Do not understand the question ☐	*Sample laboratory report*	*On-site* ☐ Off-site ☐
		**Answer**	**Verifiers**	**Comments/Additional information**
**14.a**	If your HP produces lab data, is this shared with the AMS committee, clinical teams/IPC programmes, and/or AMS programmes, or guideline development groups	Yes ☐<br>No ☐<br>Somewhat ☐<br>Unknown ☐<br>Not applicable ☐<br>Do not understand the question ☐		

**15**	Are there information technology services, tally cards or other inventory control tools available that can be used to support data gathering to support AMS activities?	Yes ☐<br>No ☐<br>Somewhat ☐<br>Unknown ☐<br>Not applicable ☐<br>Do not understand the question ☐	*Availability of functional IT services, inventory control tools*	
**16**	Are there standardised prescription charts, medical records/patient folders and transfer notes to support treatment and AMS activities?	Yes ☐<br>No ☐<br>Somewhat ☐<br>Unknown ☐<br>Not applicable ☐<br>Do not understand the question ☐	*Availability of prescription charts and medical records*	*Please report on all available*
**16.a**	Does the healthcare facility have a written policy that requires prescribers to document the indication and antibiotics prescribed in a prescription chart/medical records?	Yes ☐<br>No ☐<br>Somewhat ☐<br>Unknown ☐<br>Not applicable ☐	*Policies on prescribing*	

		Do not understand the question ☐		
**Education and Training**				
		**Answer**	**Verifiers**	**Comments/Additional information**
**17**	Does the healthcare facility include basic training in optimising antibiotic use for healthcare professionals, e.g., how to optimise prescribing, dispensing and administration in the staff induction training?	Yes ☐<br>No ☐<br>Somewhat ☐<br>Unknown ☐<br>Not applicable ☐<br>Do not understand the question ☐	*Induction training manuals*	
**18**	Does the healthcare facility offer continuous in-service training or continuous professional development on AMS and IPC to staff?	Yes ☐<br>No ☐<br>Somewhat ☐<br>Unknown ☐<br>Not applicable ☐<br>Do not understand the question ☐	*In-service training manuals/CPD/continuous medical education*	*Please specify type and regularity of training*
**19**	Does the healthcare facility ensure training for AMS team/local champion on antimicrobial stewardship/	Yes ☐<br>No ☐	*Training reports, interviews with staff*	

	infection prevention and control?	Somewhat ☐<br>Unknown ☐<br>Not applicable ☐<br>Do not understand the question ☐		
		**Answer**	**Verifiers**	**Comments/Additional information**
**20**	Did the facility hold any World Antimicrobial resistance Awareness Week (WAAW) activities in November last year?	Yes ☐<br>No ☐<br>Somewhat ☐<br>Unknown ☐<br>Not applicable ☐<br>Do not understand the question ☐	*Share some examples of activities conducted*	
**21**	Does the facility have any awareness campaigns for patients on the responsible/rational use of antibiotics?	Yes ☐<br>No ☐<br>Somewhat ☐<br>Unknown ☐<br>Not applicable ☐<br>Do not understand the question ☐		

22	Were any of the following campaign days promoted during the previous year: World Water Day, World Toilet Day, World Children's Day, Immunization campaigns, World AIDS Day, World TB Day, World Malaria Day, World Nutrition Week, for sustained action?	Yes ☐<br>No ☐<br>Somewhat ☐<br>Unknown ☐<br>Not applicable ☐<br>Do not understand the question ☐		*Please provide details*
**Monitoring and Surveillance**				
		**Answer**	**Verifiers**	**Comments/Additional information**
23	Are regular prescription audits and point prevalence surveys used **to assess the appropriateness of antimicrobial prescribing?**	Yes ☐<br>No ☐<br>Somewhat ☐<br>Unknown ☐<br>Not applicable ☐<br>Do not understand the question ☐	*Number of audits conducted with report*	
23.a	If prescription audits are undertaken, are the data used to identify AMS interventions at the facility by the AMS committee or relevant team?	Yes ☐<br>No ☐<br>Somewhat ☐		

		Unknown ☐<br>Not applicable ☐<br>Do not understand the question ☐		
**24**	Does the healthcare facility regularly monitor the quantity and type of antimicrobial use (purchased/prescribed/ dispensed)?	Yes ☐<br>No ☐<br>Somewhat ☐<br>Unknown ☐<br>Not applicable ☐<br>Do not understand the question ☐	*Antimicrobial consumption report*	
		**Answer**	**Verifiers**	**Comments/Additional information**
**24.a**	Does the healthcare facility regularly monitor shortages/stock outs of essential antimicrobials?	Yes ☐<br>No ☐<br>Somewhat ☐<br>Unknown ☐<br>Not applicable ☐<br>Do not understand the question ☐	*Stock out report*	

**24.b**	Does the healthcare facility have a mechanism for reporting substandard and falsified medicines and diagnostics?<br>*This could be an internal reporting system or direct reporting to the national system.*	Yes ☐<br>No ☐<br>Somewhat ☐<br>Unknown ☐<br>Not applicable ☐<br>Do not understand the question ☐	*Reports of substandard /falsified antimicrobials and diagnostics*	
**24.c**	Are cases of substandard and falsified medicines actually being reported at the facility?<br>*If a reporting mechanism exists, is it actively and effectively used?*	Yes ☐<br>No ☐<br>Somewhat ☐<br>Unknown ☐<br>Not applicable ☐<br>Do not understand the question ☐		
**25**	Is antimicrobial susceptibility and resistance rate for a range of key indicator bacteria regularly monitored within the facility?	Yes ☐<br>No ☐<br>Somewhat ☐<br>Unknown ☐<br>Not applicable ☐	*Antimicrobial surveillance report*	

		Do not understand the question ☐		
		**Answer**	**Verifiers**	**Comments/Additional information**
**26**	Does the AMS team monitor compliance with at least one specific AMS intervention (e.g., indication captured in medical records for patients) at the healthcare facility?	Yes ☐<br>No ☐<br>Somewhat ☐<br>Unknown ☐<br>Not applicable ☐<br>Do not understand the question ☐	*AMS intervention report*	
**Reporting Feedback within the healthcare facility**				
**27**	Does the AMS committee/team review, analyse and report on the quantities of antibiotics purchased/prescribed/dispensed to prescribers and healthcare facility management?	Yes ☐<br>No ☐<br>Somewhat ☐<br>Unknown ☐<br>Not applicable ☐<br>Do not understand the question ☐	*Antimicrobial consumption report*	
**28**	Does the AMS committee/team review, analyse and report on antibiotic susceptibility rates	Yes ☐<br>No ☐	*Evidence of dissemination of susceptibility report*	

	and key findings to prescribers?	Somewhat ☐<br>Unknown ☐<br>Not applicable ☐<br>Do not understand the question ☐		
		**Answer**	**Verifiers**	**Comments/Additional information**
**29**	Does the AMS committee/team communicate findings from audits/reviews of the quality/appropriateness of antibiotic use to prescribers, along with specific action points?	Yes ☐<br>No ☐<br>Somewhat ☐<br>Unknown ☐<br>Not applicable ☐<br>Do not understand the question ☐	*Reports to prescribers with action points*	
**29.a**	How long after data collection are findings reported back to prescribers?	N/A	*Reports to prescribers with action points*	
**30**	Does the healthcare facility develop an aggregate antibiogram (annex VIII, page 71 of WHO AMS Toolkit) and regularly update it?	Yes ☐<br>No ☐<br>Somewhat ☐<br>Unknown ☐	*Availability of Antibiogram*	

		Not applicable ☐<br>Do not understand the question ☐		

Opportunities and threats to AMS within healthcare facility and Action Plan achievements

Please provide suggested examples of initiatives to improve AMS (max 5 key actions).	1.<br>2.<br>3.<br>4.<br>5.
What do you believe are the main barriers (if any) to effective AMS in your organization?	
What are the opportunities?	

Quantitative data collection instrument 3: Narrative report (Quarterly)

The Fleming Fund | UKaid from the British people | Commonwealth Pharmacists Association | THET Partnerships for Global Health

CwPAMS 2 Narrative Report

Reporting Period: 1st April – 30th September 2023

Guidance for Health Partnerships

Please complete this narrative report for the reporting period 1st April - 30th September 2023. The intention of this report is to capture all the work that partnerships are doing outside of the MEL plan and identify areas where we can support you further. As such, the focus is on qualitative responses, as your MEL plan captures quantitative responses.

You will report against the MEL plan in our online portal. Please see the reporting webinar recording for more details or contact your grant manager if you do not know how to login to the portal.

The deadline for all reports (narrative, finance and MEL Plan data) is **20th October 2023**. Please submit your finance and narrative reports via email to your grant manager. Please complete the MEL Plan in the Portal.

1 Reporting questions

1.1 Project team

Have there been any important changes in your project team?

Please enter your answer here. This box will expand as you type.

Please list the names of people who have contributed to writing this report. This should include staff and volunteers within the UK and LMIC healthcare organisations.

- Name and position
- Organisation
- Contact email

(Please ensure that the additional contributors beyond the partners listed above (e.g., UK volunteers) have consented to their contact details being shared with THET and CPA.)

Please enter your answer here. This box will expand as you type.

1.2 Volunteer placements and surveys.

- We ask that all volunteers who have finished their time volunteering with your partnership as part of the CwPAMS project complete a volunteer survey at this link: **https://forms.office.com/e/VLpewgMWcr**https://forms.office.com/e/VLpewgMWcr. This should only be completed by volunteers who have finished volunteering during this reporting period. It can be completed by those who finish in the future at future reporting periods or at the end of the programme.

☐Have any volunteers finished their placements in the last six months?

☐If yes, have they completed the online post-placement survey?

1.3 Project Activities

Progress against activity plan with notes

Please indicate progress as follows:
Y = progress as planned

C = activity cancelled

N = activity delayed or postponed

E = activity extended

Please add in any additional activities since the partnership submitted its application. Hubs should specify activities being undertaken in each of the spokes. As a reminder, grants funded under Category A are required to undertake the following activities as a minimum, and these should be included in your activity plan:

- Sign a Memorandum of Understanding (MoU) for future work together (if not already in place).
- Develop the capacity of health workers, including pharmacists, in AMS. *See CwPAMS Tools and Resources*
- Liaise with Fleming Fund Country/Regional/Global grantees, laboratories and the fellowship scheme. E (activity extended).
- Coordinate with national AMS Committees or their equivalent to explore the take-up of policy and best practice amongst national level stakeholders. Y (progress as planned).
- Conduct and feedback data on at least one Point Prevalence Survey (PPS). E (activity extended).
- Using the CwPAMS AMS Assessment Tool, conduct and submit a baseline assessment on antimicrobial stewardship for each target institution, to inform the AMS action plan. This will be repeated towards the end of the programme. N.B., this may be provision of an existing document (CwPAMS Extension grant holders) if there have been no subsequent changes/developments since the last review. New target institutions will require an assessment. Y (progress as planned).
- Develop/update and implement a 2-3 year AMS Action Plan at each target institution, taking into consideration AMS assessment and PPS, with support from CPA. Action plans should consider possible AMS interventions and the use of relevant CwPAMS tools and resources. Y (progress as planned).

Activity	Implementation site/s	Q1	Q2	Q3	Q4	Q5	Q6	Q7

1.4 Notes on unforeseen/unplanned/cancelled activities

Please note any unplanned or unforeseen activities conducted, how they came about and how they contributed to the project objectives? Please also include an explanation for any cancelled activities.

(Max. 300 words)

Please enter your answer here. This box will expand as you type.

2 Project results

This section asks for narrative responses to progress against activities. You will report against your MEL indicators in the portal.

2.1 Challenges encountered

Please note any challenges you have encountered which have affected your ability to deliver this project. (Max. 300 words)

This may include:

- Challenges working as a partnership team.
- Impact on project activities, particularly with respect to changed timelines.
- Challenges that you have faced collecting data or measuring the impact of your project (Monitoring, Evaluation, and Learning (MEL).
- Please outline whether you need further support.

Please enter your answer here. This box will expand as you type.

2.2 Partnership and project achievements and highlights

Please summarise below any other significant progress, anticipated or not, which you have observed during this reporting period that relates to the project objectives. You may also like to comment on developments within your partnership here too. (Max. 300 words)

Please enter your answer here. This box will expand as you type.

2.3 During this reporting period, if you have provided training, please describe the type of training and how the learning outcomes were assessed against the subjects in the table below:

**Subject of Training**	**Type of training (e.g., face-to-face workshop, virtual workshop, etc.)**	**Learning outcomes assessed**

AMS		
IPC		
AMR awareness		
PPS		
Continuous Quality Improvement (CQI)		
One Health		
Use of clinical microbiology data		
Sub-standard and falsified medicines		

2.4 During this reporting period, has your partnership conducted any awareness raising activities or interventions?

**Subject of Awareness raising activity or intervention.**	**Type of intervention**	**Target Group (General Public, Patients, veterinary practitioners, community pharmacists, agriculture or food organisations, etc.)**
AMS		
IPC		
AMR awareness		
PPS		
Continuous Quality Improvement (CQI)		
One Health		

Use of clinical microbiology data		
Sub-standard and falsified medicines		

2.5 Data and Learning

	Yes/No	Further Information (Maximum 100 words)
Have you developed/revised/adopted any AMS or IPC guidelines/tools/protocols into your institutions since the start of the CwPAMS 2 programme?<br><br>*If yes, please provide details of your implementation plans for using them effectively.*		
Have you shared any PPS data with an MTC or AMS committee?<br><br>*If yes, has this resulted in any stewardship interventions?*		
Do you share information about the outcomes of your CwPAMS project with national stakeholders?<br><br>*If yes, please describe to mechanism for sharing data; if no, please describe the barriers and discuss them with your ICC.*	Yes	
During this reporting period, have you reported data specific to substandard and falsified antimicrobial medicines?<br><br>*If yes, how many sites have you done this for?*		

Have you shared any laboratory data with clinical/IPC teams?<br><br>**If yes, please describe the processes developed/established.*		
Have you shared any laboratory data with AMS committees/MTC?<br><br>**If yes, please describe the processes developed/established.*		
**Processes may include, but are not limited to, development of antibiograms and development of local guideline on integration and use of lab data. Please contact CPA for support if you are trying to develop this and are experiencing barriers.*		

2.6 If the PPS methodology (WHO/GPPS) you used during this reporting period was different from the methodology you told CPA you would use, please state this here.

Please enter your answer here. This box will expand as you type.

2.7 AMS Committees

Please provide brief examples of how your AMS committee has developed during this reporting period. This could include a list of activities. (Max. 300 words)

2.8 A fully operational AMS committee is considered to be one which has Terms of Reference, a governance reporting system, and meets regularly and can demonstrate regular actions/outputs from the meetings.

Please enter your answer here. This box will expand as you type.

- …
- …
- …
- …
- …

2.9 Hub-and-spoke evaluation.

	Yes/No	Further Information (Maximum 100 words)

Do you have experts to develop the resources/materials/interventions which you would be delivering through the hub-and-spoke model?		
Do you have a designated person/team responsible for the implementation of the interventions/actions/activities through the hub?		
Do you have enough infrastructure (equipment, software, buildings etc.) in place to support the activities/interventions/actions you are proposing<br><br>*If yes, please provide details of each infrastructural component/element.*		
Do you have data collection tools available to measure the effectiveness/progress of the interventions/activities/actions?<br><br>*If yes, please specify. If no, please provide details of the development plan (e.g., pre/post knowledge assessment for a training).*		

2.10 Please provide brief examples of activities/plans undertaken by Hub institutions to support activities and interventions amongst 'spokes'/institutions, during this reporting period. Please name the spoke sites/institutions involved (Max. 300 words)

Please enter your answer here. This box will expand as you type.

2.11 Kindly share any challenges the hub institution or team faced that have impacted the capability to carry out or advance activities, plans, and/or actions amongst 'spokes'/institutions, during this reporting period. (Max. 300 words)

Please enter your answer here. This box will expand as you type.

Please outline whether you need further support.

Please enter your answer here. This box will expand as you type.

2.12 Gender, Equity and Social Inclusion (GESI)

THET are particularly keen to understand how the projects that we are supporting are paying attention to issues of equity, especially access to training and services for vulnerable groups who are at risk of exclusion within the context.

Please describe your partnerships progress (or not) against your GESI objective(s). We would be interested to hear what is working, as well as any difficulties you have encountered so far.

Please use this section to add any further examples, feedback or details regarding gender equality and social inclusion issues (GESI). (Max. 300 words)

Please enter your answer here. This box will expand as you type.

2.13 Policy

We are interested to know how the Health Partnerships involved in CwPAMS have influenced policy in the LMIC context, beyond the institutions or implementation sites. Please reflect on the following questions and respond where appropriate for your partnership in the last six months. (Max. 300 words)

- Has your partnership developed any policies on AMS/IPC? Have these been disseminated to national stakeholders/policy makers, and how?
- Have any of the project activities resulted in a change in legislation or a policy for AMS or IPC beyond your implementation sites?
- Have any of the project activities had an impact on how pharmacists, or any other health workers involved in CwPAMS, have been recognised? Please provide an example if so.
- Have any of the project activities resulted in a change in behaviour of senior stakeholders involved in the project (e.g., hospital management or MTC members etc.)? If yes, how?

Please enter your answer here. This box will expand as you type.

3 Finance

If you would like to share any brief commentary on the overall financial report that is not already explained in the finance report document, please do so below. (Max. 200 words)

Please enter your answer here. This box will expand as you type.

4 Reporting Checklist

	Yes/No/ Not Applicable	Further details

Please submit with this report any pre-AMS Assessment Tools for any **new** sites/spokes onboarded during this period (April–September 2023).		
Please list any updated AMS Action Plans for existing institutions or newly developed AMS Action plans for spoke sites/institutions during this reporting period.<br><br>Please attach a copy with this report.		
Has the Prescribing Companion app (based on each of the country's national guidelines) been shared with/cascaded across all partnership sites and shared with any relevant in country partners?		
Have you developed any institutional based guideline that would be suitable for consideration to host on the Prescribing Companion App?<br><br>Please submit a copy of the specific guideline with this report.		

•

5 THET and CPA's Oversight

4.1 Which of the following tools and tasks have you used or completed?

Tool	Tick if used
Hub-and-spoke Assessment	
Hub-and-spoke Almanac	
Pre- and Post-Training Assessment Form	
Survey of SF medicines	
Prescribing Companion App	
AMS board game	
CwPAMS toolkit: AMS Toolkit	
CwPAMS Behavioural Science Toolkit for AMS (and Tips for Communication; TCE Report)	
AMS animation explainer videos	

CPD Platform- Behavioural change module	
CPD platform – antimicrobial stewardship module	
How to Manufacture Alcohol Hand Rub: Training video	
Knowledge Attitudes and Behaviours survey template	
Publications	
Please add any other CwPAMS resources used that are not listed here.	

4.2 What kind of support from THET and CPA has been the most useful and why? What type of support would be useful for the rest of the programme? (Max. 300 words)

Please enter your answer here. This box will expand as you type.

The fellowship programme, ALF-A, has been very useful for capacity building in project management and leadership. The fellowship is improving our knowledge on project management, hence smooth running of the project so far.

The clear support and direction for the finalisation of MEL plans have been helpful.

5 Communications

In this reporting period, have you produced any publications related to CwPAMS 2? If yes, please provide the link to the publication and any social media posts used to disseminate the publication/s.

Please enter your answer here. This box will expand as you type.

We would like to showcase some highlights on Pulse of the work CwPAMS 2 partnerships are carrying out over the course of the programme. Please share with us one highlight from your project over the last six months. If you have written a blog or social media post, please feel free to share the hyperlink below and/or any photos.

Please note: THET and CPA uses photographs and quotes to communicate messages and document the work of projects. These images and quotes become a valuable resource, not only for us, but for Health Partnerships, our supporters and potential donors. These images appear on our website, social media sites, Fops and at events. Our Code of Conduct for Ethical Storytelling sets out how photographs and videos of people involved in our activities should be taken and shared. You can find this Code of Conduct in your welcome pack or on Pulse.

THET and CPA believes in upholding the rights of its contributors to participate and be heard, to experience respect and dignity, to make free and informed decisions, and to be protected from risk and harm. We believe that every person – child, young person, and adult – must be consulted about the use of their image and must give consent to it being used and shared. THET and CPA recognise the need to ensure the welfare and safety of all people whose images we use.

Data collection instrument 4: SF MEDS Knowledge, attitude and perception survey

SECTION A: SOCIO DEMOGRAPHICS

Please select and fill in with the most appropriate information.

1. Gender

- Male
- Female
- Prefer not to answer

2. Age

- 20 - 29
- 30 - 39
- 40 - 49
- 50 - 59
- > 60

3. **Highest degree completed**

- Certificate
- Diploma
- Bachelor's degree
- Master's degree
- Doctoral Degree
- Other (Please specify) ……………………

4. Professional background (select more than one if needed)

- Pharmacy
- Medicine
- Nursing
- Midwifery
- Laboratory
- Other area of practice (Please specify) ……………………

5. Number of years in service in a health setting

- <1 year
- 1-5 years
- 6-10 years
- 11-15 years
- 16-20 years

- >20 years

6. Type of facility
- Public
- Private
- Private Not for Profit
- Other (Please specify) ……………………

7. Health Facility Level
- National Referral Hospital
- Regional Referral Hospital
- General Hospital (District)
- Health Center IV
- Health Center III
- Health Center II
- Other (Please specify) ……………………

8. Country of Practice
Drop down list of the 8 countries

SECTION B: KNOWLEDGE OF SUBSTANDARD AND FALSIFIED MEDICINES
This section will assess your knowledge on Substandard and Falsified Medicines (SFMs)

9. Do you believe that SFMs are available in your country of practice?
- Yes
- No
- Don't know/Not sure

10. How best would you describe Substandard or Falsified Medicines? (Select all that apply)
- Authorised pharmaceutical products that fail to meet quality standards or specifications (or both) and originate from poor manufacturing practices; supply chain gaps or inappropriate storage that lead to degradation of the active ingredients.
- Illicit pharmaceutical products that result from deliberate/fraudulent misrepresentation of the identity, composition or source.
- Others (please specify)……………………

11a. Which of the following would be considered a substandard medicine? (Select all that apply)
- Medicine deliberately produced without the active ingredient
- Medicine deliberately produced using the wrong active ingredient
- Medicine deliberately containing ingredients of bad/toxic quality
- Medicine deliberately and fraudulently mislabelled
- Medicine with the wrong amount of the right active ingredient (may contain toxic doses)
- Medicine containing impurities/contaminants (may contain toxic doses)

- Medicine that compromises patient safety (may cause mass poisoning or result in a fatal outcome for the patient)

g3[illegible] **Which of the following would be considered falsified medicine? (Select all that apply.)**

- Medicine deliberately produced without the active ingredient
- Medicine deliberately produced using the wrong active ingredient
- Medicine deliberately containing ingredients of bad/toxic quality
- Medicine deliberately and fraudulently mislabelled
- Medicine with the wrong amount of the right active ingredient (may contain toxic doses)
- Medicine containing impurities/contaminants (may contain toxic doses)
- Medicine that compromises patient safety (may cause mass poisoning or result in a fatal outcome for the patient)

12. Which of these therapeutic categories could be substandard and falsified in your country of practice? (Select all that apply)

- Medicines
- Vaccines
- In vitro diagnostics
- Don’t know/Not sure
- Other (Please specify)……………………

13. What immediate effects do you think are associated with SFMs? (Select all that apply)

- Increased morbidity
- Serious adverse reactions
- Poisoning
- No therapeutic response
- Death
- Don’t know/Not sure
- Other (Please specify) ……………………

14. What mid/long-term effects can be associated with SFMs? (Select all that apply)

- Antimicrobial resistance
- Increase out-of-pocket spending
- Lost productivity
- Lost income
- Slow poisoning
- Death
- Don’t know/Not sure
- Other (Please specify) ……………………

SECTION C: DETECTION OF SUBSTANDARD AND FALSIFIED MEDICINES (SFMs)

This section will assess your knowledge of detection Substandard and Falsified Medicines (SFMs)

15. What are the target sites for SFMs in your country of practice? (Select all that apply)

- Hospitals
- Pharmacies

- Clinics
- Retail drug shops
- Online resources
- Don’t know/Not sure
- Other (Please specify) ……………………

16. Where can you find SFMs in the distribution system in your country of practice?
- Unregulated websites
- Illegal street markets
- Health centers
- Hospitals
- Clinics
- Pharmacies
- Drug shops
- Don’t know/Not sure
- Other (Please specify) ……………………

17. How likely do you think you would be able to identify SFMs?
- Extremely Likely
- Likely
- Neutral
- Unlikely
- Extremely Unlikely

18. How can you detect SFMs? (Select all that apply)
- Examining the packaging for condition, spelling mistakes or grammatical errors
- Checking the manufacture and expiry dates and ensuring any details on the outer packaging match the dates shown on the inner packaging
- Ensuring the medicine looks correct, is not discolored or degraded, or has an unusual smell
- Don’t know/Not sure
- Other (Please specify) ……………………

19a. Have you encountered SFMs in your general practice in the past one year?
- Yes
- No
- Don’t know/Not sure

19b. If yes, what was the classification of those SFMs?
- Vaccines
- Antibiotics
- Antivirals
- Antimalarials
- Other anti-parasitics
- Contraceptives
- Other (Please specify) ……………………

20a. Are there any barriers to SFM detection in your country?
- Yes
- No
- Don't know/Not sure

20b. If yes, please provide details and/or examples of those barriers.
……………………………………………………………………………

SECTION D: REPORTING OF SUBSTANDARD AND FALSIFIED MEDICINES

21a. Are there any mechanisms/reporting channels in your country for SFMs?
- Yes
- No
- Don't know/Not sure

21b. If yes, at what level are the mechanisms?
- Local
- Regional
- National
- Other (Please specify) ……………………

21c. If yes, what mechanisms are these?
……………………………………………………………………………

22a. Are there any barriers to SFMs reporting in your country of practice?
- Yes
- No
- Don't know/Not sure

22b. If yes, please provide details and/or examples of barriers.
……………………………………………………………………………

SECTION E: SUPPORT ON SUBSTANDARD AND FALSIFIED MEDICINES

23a. Have you ever attended any training on SFMs?
- Yes
- No

23b. If yes, what was the training about?
……………………………………………………………………………

24a. Would you like further educational support on SFMs? (Please select all that apply)
- Yes, I would like further educational support to be able to detect (or identify) SFMs.
- Yes, I would like further educational support to be able to report SFMs.
- Any other support (Please specify) ……………………

- No

24b. If yes, how would you want the support to be delivered? (Please select all that apply)

- Webinars
- Training (e.g., course, workshop)
- Self-directed learning
- Video-graphics/animation videos
- Other (Please specify) ……………………

25. Do you have any other comments?

………………………………………………………………………
